# Utility of interferon gamma/tumor necrosis factor alpha FluoroSpot assay in differentiation between active tuberculosis and latent tuberculosis infection: a pilot study

**DOI:** 10.1186/s12879-021-06351-w

**Published:** 2021-07-06

**Authors:** Lifan Zhang, Shijun Wan, Ziyue Zhou, Yueqiu Zhang, Xiaoqing Liu

**Affiliations:** 1 Division of Infectious Diseases, Peking Union Medical College Hospital, Chinese Academy of Medical Sciences & Peking Union Medical College, Beijing, 100730 China; 2 Clinical Epidemiology Unit, International Epidemiology Network, Peking Union Medical College Hospital, Chinese Academy of Medical Science, Beijing, 100730 China; 3 Centre for Tuberculosis Research, Chinese Academy of Medical Sciences and Peking Union Medical College, Beijing, China; 4 Peking Union Medical College Hospital, Peking Union Medical College, Beijing, China

**Keywords:** Tuberculosis, Latent tuberculosis infection, Tumor necrosis factor, Interferon gamma, FluoroSpot, Diagnosis

## Abstract

**Background:**

The differential diagnosis of active tuberculosis (ATB) and latent tuberculosis infection (LTBI) remains challenging in clinical practice. We aimed to evaluate the diagnostic accuracy of the IFN-γ/TNF-α FluoroSpot assay for differentiating ATB from LTBI.

**Methods:**

We conducted a pilot study of case-control design, using the FluoroSpot assay to simultaneously detect IFN-γ and TNF-α secretion at the single-cell level. The frequencies of antigen-specific single TNF-α-, total TNF-α-, single IFN-γ-, total IFN-γ- and dual IFN-γ/TNF-α-secreting T cells were detected. The optimal cutoffs value of frequencies for differentiating ATB from LTBI were determined according to receiver operating characteristic curve analysis. The sensitivity, specificity, predictive values (PV) and likelihood ratios (LR) of the FluoroSpot assay were calculated.

**Results:**

Thirty patients diagnosed microbiologically with ATB, 36 healthcare workers with LTBI and 36 healthy controls were enrolled. After stimulated by ESAT-6 or CFP-10 peptides, the median frequencies of single TNF-α-, total TNF-α-, single IFN-γ-, total IFN-γ- and dual IFN-γ/TNF-α-secreting T cells in ATB patients were all significantly higher than those in LTBI and HC groups (*P* < 0.01). The frequencies of total IFN-γ-secreting T cells detected by FluoroSpot assay correlated significantly with those of T-SPOT.TB (r = 0.910 for ESAT-6, *P* < 0.001, r = 0.845 for CFP-10, *P* < 0.001). After stimulated by ESAT-6 peptides, with total TNF-α-secreting T cells frequencies at a cut off value of 21 iSFCs/250,000 PBMCs, the sensitivity, specificity, PLR, NLR, PPV, NPV of IFN-γ/TNF-α FluoroSpot assay in differentiating ATB from LTBI were 96.7% (95%CI, 82.8–99.9%), 94.3% (95%CI, 80.8–99.3%), 16.92 (95%CI, 4.40–65.08), 0.04 (95%CI, 0.01–0.24), 93.6% (95%CI,78.6–99.2%) and 97.1% (95%CI, 84.7–99.9%), respectively. With the frequencies of total TNF-α- and total IFN-γ-secreting T cells stimulated by ESAT-6 peptides combined, the specificity was increased to 97.1%, and the positive likelihood ratio to 31.5. The combination with CFP-10 might not improve the diagnostic accuracy of the ESAT-6 for differentiating ATB from LTBI.

**Conclusions:**

IFN-γ/TNF-α FluoroSpot assay might have potential to help differentiate ATB from LTBI, but the findings need to be further verified by cross-sectional or prospective cohort studies.

**Supplementary Information:**

The online version contains supplementary material available at 10.1186/s12879-021-06351-w.

## Background

Tuberculosis (TB) is known to be the leading cause of death from a single infectious pathogen. Worldwide 10.0 million people were newly diagnosed with TB disease, and TB caused 1.2 million deaths among HIV-negative people in 2019 [1]. China has the second largest number of TB cases in the world, accounting for 8.4% of all TB cases [1]. Timely diagnosis and successful treatment could prevent millions of TB deaths each year, but currently there isn’t an ideal test for diagnosing TB rapidly and accurately, especially for extrapulmonary TB.

WHO defined LTBI as a state of persistent immune response to stimulation by *Mycobacterium. tuberculosis* (MTB) antigens with no evidence of clinically manifest ATB [2]. It is estimated that about 23% (1.7 billion) of the global population have latent TB infection [3]. Tuberculin skin test (TST) and Interferon-gamma release assay (IGRA) are commonly used to identify TB infection. However, the cross-reactive immune response between purified protein derivative (PPD) and Bacille Calmette-Gue’rin (BCG) may lead to false positive TST results, which may greatly reduce the specificity of TST in China, where BCG vaccination was included in the Newborn Immunization Schedule [4]. IGRA diagnosed TB infection by detecting IFN-γ released by MTB-specific antigen stimulation. However, it cannot distinguish ATB with LTBI [5]. Hence, new methods that can accurately differentiate ATB and LTBI are imperative for countries with high TB disease burden like China.

Tumor necrosis factor (TNF) participates in MTB immune response by activating macrophages, inducing chemokines, participating in TB granuloma formation and maintaining granuloma integrity. In addition, animal experiments showed that blocking the TNF signaling pathway accelerated intracellular bacterial growth and necrotic death of overladen macrophages, implicating that TNF restricts mycobacterial growth within macrophages and prevents their necrosis [6]. Human studies demonstrated that patients with rheumatic disease using TNF-α antagonists had an increased risk of ATB and LTBI reactivation [7], suggesting that TNF-α is a key factor in controlling MTB invasion and proliferation. Based on these studies, we hypothesize that the combination of IFN-γ and TNF-α may be helpful in the differential diagnosis of ATB and LTBI.

FluoroSpot is a highly sensitive and practical technique based on ELISPOT, which simultaneously detect two or more cytokine secretions at single-cell level. The interpretation of the results is based on the visualization of the cells with distinct fluorescence, thus avoiding color confusion [8, 9]. FluoroSpot is found to have high consistency with ELISPOT [9]. We aim to evaluate the diagnostic accuracy of IFN-γ/TNF-α FluoroSpot assay in differentiating ATB from LTBI, with microbiology as the reference standard.

## Participants and methods

The study was conducted in accordance with the Declaration of Helsinki and was approved by the Ethics Committee of PUMCH (No: S-715). Informed written consent was obtained from all patients prior to their enrollment in this study.

### Participants

From March 2018 to September 2018, ATB patients and healthcare workers were enrolled from Peking Union Medical College Hospital and Beijing Chest Hospital. The inclusion criteria for the active TB group were: 1) aged from 18 to 75 years old, AND 2) had typical clinical manifestations of ATB such as cough, chest pain, night sweats, weight loss, etc. AND 3) ATB diagnosis made based on microbiological evidence (including sputum smear-positive, positive MTB culture, positive Nucleic Acid Amplification (NAA) testing or Xpert MTB/RIF assay), AND 4) had not received any anti-TB therapy by the time of recruitment. The inclusion criteria for healthcare workers were: 1) aged from 18 to 75 years old, AND 2) without clinical manifestations suspected of ATB, AND 3) without evidence of previous TB such as self-reported TB history or positive X-ray findings, AND 4) without autoimmune diseases, diabetes mellitus, chronic hepatitis or malignancy. Healthcare workers with positive and negative T-SPOT.TB testing results were regarded as LTBI and healthy controls (HC), respectively. Persons who were pregnant, receiving anti-TNF-α therapy, or infected with HIV were excluded. This study was a case-control designed diagnostic accuracy study.

### IFN-γ/TNF-α FluoroSpot assay

**Fig. 1:**
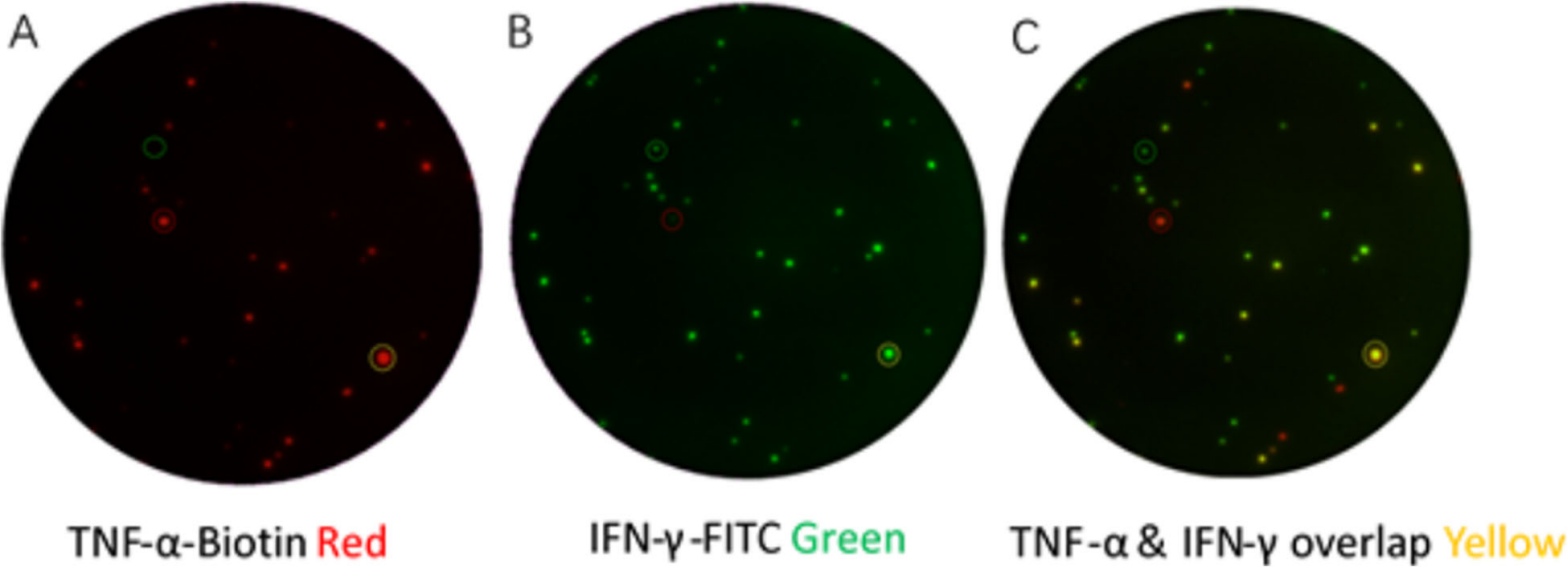
MTB-Specific TNF-α-, IFN-γ- and dual IFN-γ/TNF-α-secreting T cells detected with FluoroSpot assay. Images of a well captured under different filters are presented as an example. (**A**) Red spots represent total TNF-α specific T cells. (**B**) Green spots represent total IFN-γ specific T cells. (**C**) Overlay of (**A**) and (**B**). Yellow spots from superposition of red and green fluorescence represent T cells dually secreting IFN-γ and TNF-α after stimulation. Single cytokine (TNF-α or IFN-γ) secreting T cell frequencies equals the frequencies of dual secreting T cells subtracted from total T cells

Four milliliters of peripheral blood were obtained from each participant by venipuncture. Within 4 h of sample collection, peripheral blood mononuclear cells (PBMCs) were isolated from heparinized whole blood by density gradient centrifugation. Cell suspension was prepared at 2.5 × 10^6^ PBMCs/ml with serum free culture medium (Gibco™ AIM V Medium liquid, Invitrogen, USA, abbreviated as AIM-V). IFN-γ/TNF-α FluoroSpot (Mabtech AB, Sweden) assays were performed according to the manufacturer. 96-well plates pre-coated with monoclonal antibodies against IFN-γ and TNF-α were seeded with 2.5 × 10^5^ PBMCs and anti-CD28 (at 0.1 μg/ml, Mabtech AB, Sweden). 50 μl of AIM-V (Gibco™ AIM V Medium liquid, Invitrogen, USA) as nil control, 5 μg/ml PHA as positive control, and 6-kDa early secreted antigenic target (ESAT-6) and 10-kDa culture filtrate protein (CFP-10) peptides (final concentration at 10 μg/ml of each peptide) as MTB-specific antigens were added in duplicate wells. All peptides were 15mers overlapping their adjacent peptides by 10 amino acids, spanning the length of ESAT-6 (17 peptides) or CFP-10 (18 peptides). The purity of each peptide exceeded 80%, and identity was confirmed by mass spectrometry. Plates were incubated at 37 °C in 5% carbon dioxide for 16–20 h and then washed with washing buffer. After incubation, the detection antibodies (anti-TNF-α biotin and anti-IFN-γ-FITC) were added to each well and incubated for two hours at room temperature under dark condition. Then streptavidine-550 red-conjugate and anti-FITC-490 green were added to the reaction system. The reaction system was incubated for another one hour under dark and dry condition. Finally, fluorescence enhancer was loaded to make spots visible under the automated ELISPOT reader (AID iSpot, Strassberg, Germany). When counting the frequencies of ESAT-6 or CFP-10 antigen-specific immunofluorescence spot forming cells (iSFCs), including frequencies of total TNF-α- or total IFN-γ- or dual IFN-γ/TNF-α-secreting T cells, the background spots in the nil control well were subtracted. If the number of spots was over 50 iSFCs/2.5 × 10^5^ PBMC for TNF-α or over 10 iSFCs/2.5 × 10^5^ PBMC for IFN-γ in nil control, or less than 200 iSFCs/2.5 × 10^5^ PBMC in positive control, the result was considered to be indeterminate. Images of the results are presented in Fig. 1.

Laboratory staff who conducted the assays and researchers who interpreted the results were all blinded to patients’ clinical data.

### Statistical analysis

We used the histogram to check the distribution of the variables. For normally distributed variables, results were presented as mean ± standard deviation (SD), while variables with an abnormal distribution were denoted as median and interquartile range (IQR). Categorical data were presented as percentages and 95% confidence intervals (CIs). We used Mann-Whitney U test to compare the frequencies of iSFCs and Chi-square test to compare response proportions between different groups, respectively. We used Wilcoxon signed-rank test to compare the frequency of iSFCs between the two antigens in the same group. We used Spearman’s rank correlation to measure correlation between the frequencies of total IFN-γ-secreting T cells detected by FluoroSpot assay and T-SPOT.TB.

Receiver operating characteristic (ROC) curves were used to show the overall accuracy of IFN-γ/TNF-α FluoroSpot assay for differentiating ATB and LTBI in frequencies of T cells. The areas under the ROC curves (AUROC) of each group of T cells were calculated and compared. The cutoff value with the largest Youden’s index on the ROC curve was regarded as the optimal cutoff of the frequency. The sensitivity, specificity, predictive value (PV) and likelihood ratio (LR) were calculated at the optimal cutoff.

Statistical analyses were performed using SPSS 24.0 (SPSS Inc., Chicago, IL, USA) and MedCalc (version 11, MedCalc Software bvba, Mariakerke, Belgium). *P* < 0.05 (two-sided) were considered as statistically significant.

## Results

### Demographic *and clinical features of participants*

A total of 101 participants, including 30 patients with microbiologically confirmed ATB, 35 participants with LTBI and 36 healthy controls were enrolled. Of the 30 ATB patients, 23 (76.7%) were positive for MTB culture, 16 (53.3%) were positive for sputum smear acid-fast staining, 24 (80.0%) were positive for MTB-NAA testing, and 20 (66.7%) were positive for Xpert MTB/RIF test. 27 (90.0%) ATB patients were diagnosed with pulmonary tuberculosis, while tuberculous lymphadenitis and tuberculous pleuritis were diagnosed in 2(6.7%) and 1 (3.3%) patient, respectively. Detailed characteristics of the participants are shown in Table 1.

**Table 1:** Characteristics of participants

Characteristics	ATB (*n* = 30)	LTBI (*n* = 35)	HC (*n* = 36)	Total (*n* = 101)
Sex (M / F)	13/17	4/31	8/28	25/76
Age (mean ± SD)	48 ± 18	40 ± 9	36 ± 10	41 ± 13
Complications (n, %)				
Malignancy	0(0.0%)	0(0.0%)	0(0.0%)	0(0.0%)
Renal failure	0(0.0%)	0(0.0%)	0(0.0%)	0(0.0%)
Diabetes mellitus	1(3.3%)	0(0.0%)	0(0.0%)	1(1.0%)
TB history	2(6.7%)	0(0.0%)	0(0.0%)	2(2.0%)
Autoimmune diseases	1(3.3%)	0(0.0%)	0(0.0%)	1(1.0%)
Corticosteroid therapy	1(3.3%)	0(0.0%)	0(0.0%)	1(1.0%)
Immunosuppressants	0(0.0%)	0(0.0%)	0(0.0%)	0(0.0%)
Blood tests				
WBC(*10^9^/L)	6.96 ± 2.50	6.31 ± 1.63	6.51 ± 1.83	6.59 ± 2.01
Lymphocyte(*10^9^/L)	1.49 ± 0.58	2.01 ± 0.45	2.23 ± 0.63	1.91 ± 0.63

**Abbreviations:** ATB, active tuberculosis; LTBI, latent tuberculosis infection; HC, healthy controls; M, male; F, female; SD, standard deviation; TB, tuberculosis; WBC, white blood cell

### Frequencies of MTB specific IFN-γ/TNF-α-secreting T cells

**Fig. 2:**
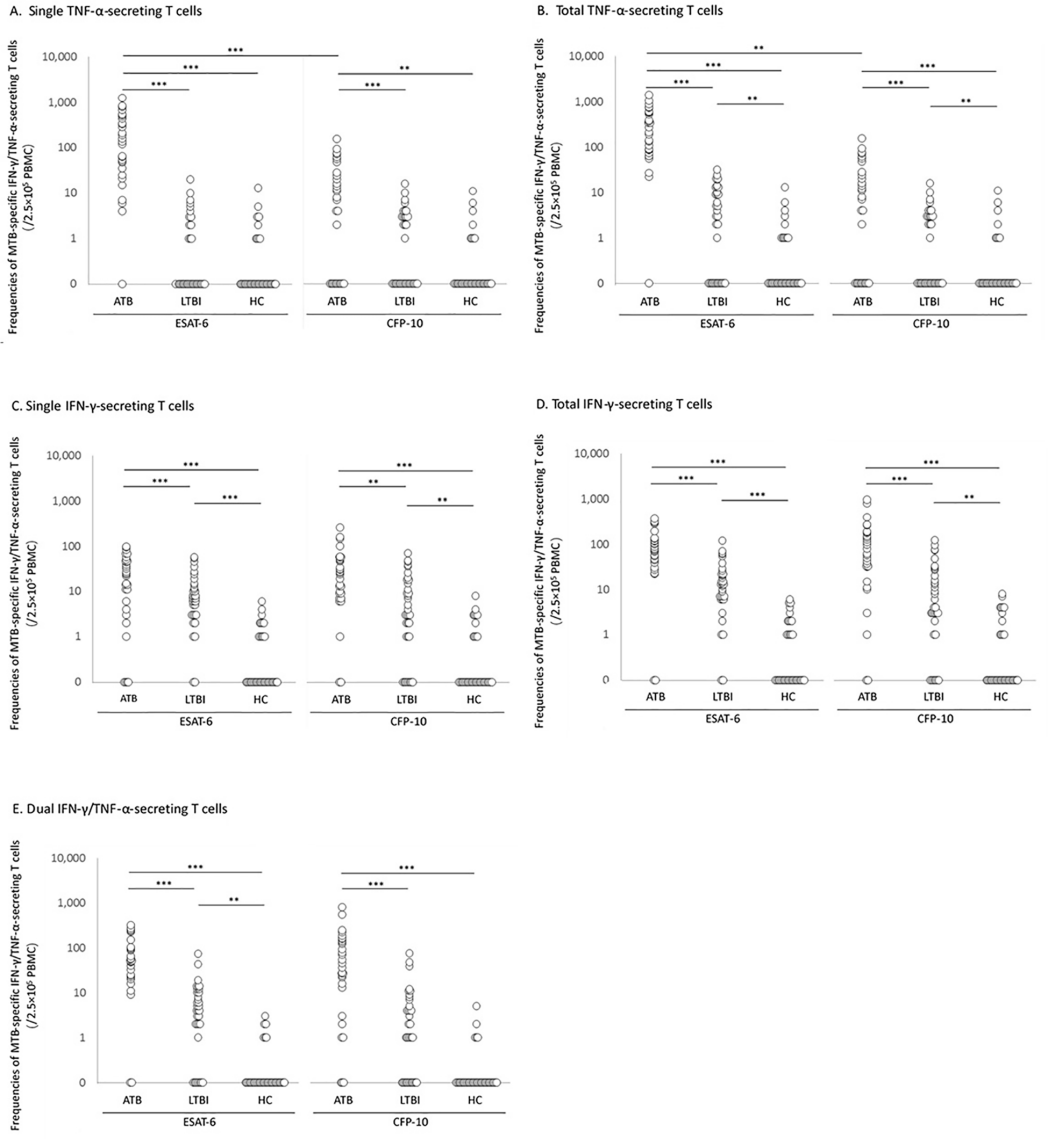
Comparison of frequencies of single TNF-α-, total TNF-α-, single IFN-γ-, total IFN-γ-, dual IFN-γ/TNF-α-secreting T cells following ESAT-6 or CFP-10 stimulation in ATB, LTBI and HC groups. T cell frequencies are estimated as per 2.5 × 10^5^ PBMC. Each point represents a sample. (**A**) Frequencies of T cells secreting only TNF-α, without IFN-γ. (**B**) Frequencies of all the T cells secreting TNF-α, with or without IFN-γ secretion. (**C**) Frequencies of T cells secreting only IFN-γ, without TNF-α. (**D**) Frequencies of all the T cells secreting IFN-γ, with or without TNF-α secretion. (**E**) Frequencies of T cells secreting both IFN-γ and TNF-α simultaneously. Abbreviations: ATB, Active tuberculosis; LTBI, latent tuberculosis infection; HC, healthy control; MTB, *mycobacterium.tuberculosis.* ***P < 0.001, **P < 0.01

After stimulated by ESAT-6 or CFP-10 peptides, the frequencies of single TNF-α-, total TNF-α-, single IFN-γ-, total IFN-γ-, dual IFN-γ/TNF-α-secreting T cells in ATB group were significantly higher than those in LTBI group and HC group (*P* < 0.01). The frequencies of total TNF-α-, single IFN-γ-, total IFN-γ-, dual IFN-γ/TNF-α-secreting T cell in LTBI group were significantly higher than those in HC group under single ESAT-6 stimulation (Fig. 2, Supplementary Table 1).

In the ATB group, the median frequencies of total TNF-α-secreting T cells was 276[IQR, 88–581] iSFCs/2.5 × 10^5^ PBMC when stimulated by ESAT-6, compared to only 109[IQR, 40–210] iSFCs/2.5 × 10^5^ PBMC stimulated by CFP-10 (*P* = 0.001). In consistency with the result, the median frequencies of single TNF-α-secreting T cells stimulated by ESAT-6 were significantly higher than those stimulated by CFP-10 (*P* < 0.001) (Fig. 2).

There was no indeterminate result of IFN-γ/TNF-α FluoroSpot assay in all 101 study samples. When counting the frequencies of TNF-α-secreting T cells in nil control wells, 38.6% (39 in 101) displayed over 10 iSFCs/2.5 × 10^5^ PBMC, and the median frequencies of TNF-α-secreting T cells in ATB, LTBI and HC groups were 24[IQR, 6–39], 4[IQR, 0–11] and 4[IQR, 1–11] iSFCs/2.5 × 10^5^ PBMC, respectively. (Supplementary Table 2).

### Comparison of MTB specific IFN-γ response between FluoroSpot assay and T-SPOT.TB

**Fig. 3:**
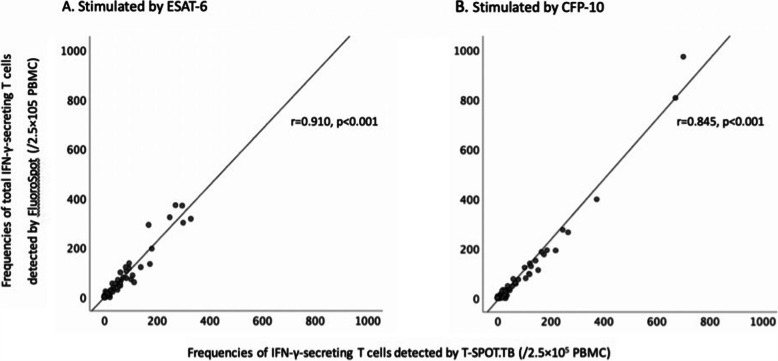
Correlation between the frequencies of total IFN-γ-secreting T cells detected by FluoroSpot assay and those of T-SPOT.TB. (**A**) Stimulated by ESAT-6. (**B**) Stimulated by CFP-10

After stimulated by ESAT-6 or CFP-10 peptides, the frequencies of total IFN-γ-secreting T cells detected by FluoroSpot assay strongly and significantly correlated with those of T-SPOT.TB (r = 0.910 for ESAT-6, *P* < 0.001, r = 0.845 for CFP-10, *P* < 0.001) (Fig. 3). The agreement between the results of FluoroSpot assay and T-SPOT.TB was almost perfect (kappa = 0.852, *p* < 0.001). In active TB group, the sensitivity of Fluorospot assay detecting total IFN-γ-secreting T cell was 93.3%, and there was no significant difference compared with T-SPOT.TB (96.7%, *P* = 1.000).

### Diagnostic accuracy of IFN-γ/TNF-α FluoroSpot assay in distinguishing ATB from LTBI

**Fig. 4:**
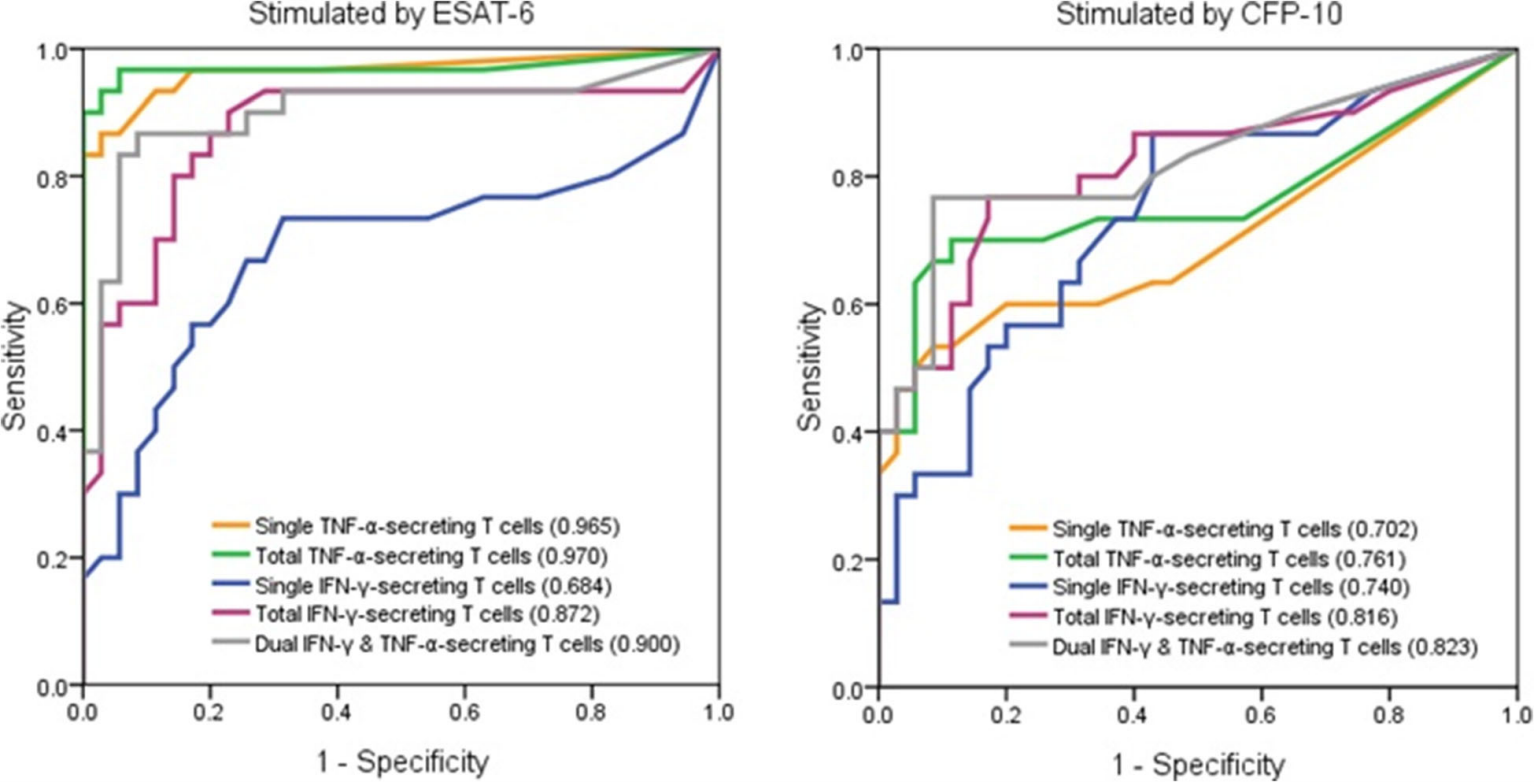
ROC curves and the corresponding AUROC for measurement of frequencies of single TNF-α-, total TNF-α-, single IFN-γ-, total IFN-γ-, dual IFN-γ/TNF-α-secreting T cells in differentiating ATB and LTBI under stimulation of ESAT-6 or CFP-10

In the ATB and LTBI groups, the AUROC of the frequencies of ESAT-6-specific total TNF-α-secreting T cell was the largest in differentiating ATB from LTBI, which was 0.970 (95%CI, 0.894–0.996) (Fig. 4). The optimal cutoff value was 21 iSFCs/2.5 × 10^5^ PBMC, with a sensitivity of 96.7% (95% CI, 82.8–99.9%) and a specificity of 94.3% (95% CI, 80.8–99.3%), respectively. The combination of ESAT-6 and CFP-10 did not improve the diagnostic accuracy of differential diagnosis of ATB and LTBI (Supplementary Table 3).

Parallel tests [10] combining the frequencies of total TNF-α- and total IFN-γ-secreting T cells (with optimal cutoff value: 23 iSFCs/2.5 × 10^5^ PBMC) improved the differential diagnostic accuracy (Table 2).

**Table 2:** **Diagnostic accuracy of the IFN-γ/TNF-α FluoroSpot assay for differentiating ATB from LTBI (Stimulated by ESAT-6 peptide)**

	Sensitivity (%, 95CI)	Specificity (%, 95CI)	PLR (95%CI)	NLR (95%CI)	PPV (%, 95CI)	NPV (%, 95CI)
Frequencies of total TNF-α-secreting T cells	96.7 (82.8–99.9)	94.3 (80.8–99.3)	16.92 (4.40–65.08)	0.04 (0.01–0.24)	93.6 (78.6–99.2)	97.1 (84.7–99.9)
Frequencies of total IFN-γ-secreting T cells	90.0 (73.5–97.9)	77.1 (59.9–89.6)	3.94 (2.12–7.32)	0.13 (0.04–0.39)	77.1 (59.9–89.6)	90.0 (73.5–97.9)
Parallel test						
With the OR rule*	96.7 (82.8–99.9)	74.3 (56.7–87.5)	3.76 (2.13–6.63)	0.04 (0.01–0.31)	76.3 (59.8–88.6)	96.3 (81.0–99.9)
With the AND rule^#^	90.0 (73.5–97.9)	97.1 (85.1–99.9)	31.50 (4.55–218.21)	0.10 (0.04–0.30)	96.4 (81.7–99.9)	91.9 (78.1–98.3)

If the frequencies of total TNF-α-secreting T cells were greater than 21 iSFCs/2.5 × 10^5^ PBMC, AND more than twice as large as that of the nil control, the result of TNF-α FluoroSpot assay was considered positive. If the frequencies of total IFN-γ-secreting T cells were greater than 23 iSFCs/2.5 × 10^5^ PBMC, AND more than twice as large as that of the nil control, the result of IFN-γ FluoroSpot assay was considered positive.

*The OR rule, in which the diagnosis is positive if either test is positive

^#^The AND rule, in which the diagnosis is positive only if both tests are positive

**Abbreviations:** ATB, active tuberculosis; LTBI, latent tuberculosis infection; ESAT-6, 6-kDa early-secreted antigenic target; PLR, positive likelihood ratio; NLR, negative likelihood ratio; PPV, positive predictive value; NPV, negative predictive value; iSFCs, immunofluorescence spot forming cells; PBMC, peripheral blood mononuclear cells

## Discussion

Conventional diagnostic methods for tuberculosis are not satisfying in clinical practice as acid-fast bacilli (AFB) smear lacks sensitivity and mycobacterial culture is time consuming [11]. Although Xpert MTB/RIF can detect MTB and rifampin-resistance mutations in several hours with high accuracy, it is estimated that 30–50% patients failed to provide appropriate samples for the test [12]. Our previous study showed that less than 30% of patients diagnosed with ATB could be confirmed by etiology [13]. Thus, clinicians urgently need new methods to help diagnose TB, especially for patients who cannot obtain evidence of the pathogens.

T-cell immunity plays a critical role in controlling MTB infection [14]. IGRA works by detecting the secretion of Th1 cytokine IFN-γ after MTB-specific antigen stimulation to diagnose TB infection [15]. In addition to IFN-γ, TNF-α is also involved in immunity against MTB infection. Secreted by activated dendritic cells, γδT cells [16], macrophages, CD4+ as well as CD8+ T cells, TNF-α attaches to endothelin cells and improves vascular permeability so that neutrophils and monocytes in circulation recruited by inflammation cytokines can enter infection sites and further form granuloma. TNF-α maintains granuloma integrity through transforming inactivated macrophage to M1 subtype, a process synergizing with IFN-γ [17]. Clinical studies observed higher reactivation and incidence of TB in patients with anti-TNF-α therapy [18–20]. Therefore, whether MTB-specific TNF-α secretion can contribute to the diagnosis of ATB is worthy of further study.

Our study showed that the frequencies of MTB specific IFN-γ-secreting T cells in the ATB group were significantly higher than those in the LTBI and HC groups. These findings were consistent with our previous research [21]. The frequencies of TNF-α-secreting T cells was comparable to the results of other studies using ELISA or flow cytometry (FCM) [22–24]. The FluoroSpot assay based on the ELISPOT is more accurate compared to ELISA or FCM. The Fluorospot assay may be preferable for studies requiring the detection of low-level responses or qualitative results [25]. In addition, compared to FCM, FluoroSpot assay enables single-cell measurements of the actual secretion of bioactive molecules rather than intracellular analytes, which can be critical for understanding functional properties of T cells, as it may help in better identification of infection status [26].

Interestingly, ESAT-6 seemed to activate T cells more effectively than CFP-10. The frequencies of TNF-α-secreting T cells stimulated by ESAT-6 were significantly higher than those stimulated by CFP-10, though no difference was detected in the IFN-γ secretion under the stimulation of these two antigens. The immune mechanism is still unclear and remains to be further studied.

In this study, the frequencies of ESAT-6-specific total TNF-α-secreting T cells might be a better potential marker for IFN-γ/TNF-α FluoroSpot assay to differentiate ATB and LTBI, compared with IFN-γ-secreting T cells. Wang’s study showed that the detection of. of MTB-specific TNF-α secretion by ELISA assay could distinguish ATB from LTBI, with a sensitivity of 72% and a specificity of 90.91% [27]. Another study evaluated the diagnostic accuracy of differentiating ATB from LTBI by combining the IGRA and the TNF-α-release assay (TARA). Compared with IGRA only, the combination of IGRA and TARA improved the specificity without compromising the sensitivity [28]. IFN-γ/TNF-α FluoroSpot assay was a new method to diagnose ATB, and only one study showed that IFN-γ/TNF-α dual release assay had the best accuracy to differentiate ATB and LTBI, with a sensitivity of 84% and a specificity of 94%. This was slightly different from our results, which might be due to the different inclusion criteria of the ATB patients. In addition to the microbiologically confirmed cases, the study also included clinically diagnosed TB cases, who possibly displayed different cytokine secretion patterns. It should also be noted that the patients’ medication was not mentioned in the study, which may affect the results [29].

Furthermore, this study showed that double-testing the ESAT-6 with the CFP-10 might not increase the differential diagnostic accuracy. The result was in consistency with a study of North India population which also observed significantly higher IFN-γ and TNF-α response to ESAT-6 stimulation in ATB group compared to healthy household contacts (considered as LTBI), while no significant difference was observed with CFP-10 stimulation [30]. Clifford’s study showed that the level of TNF-α stimulated by ESAT-6 declined significantly over the course of therapy in ATB cases, but the decrease was not significant under CFP-10 stimulation [31]. Therefore, using ESAT-6 antigen alone might reduce the cost of testing without compromising diagnostic accuracy.

Medication may disturb the cytokine secretion. Several studies of IGRAs indicated that anti-tuberculosis drugs, corticosteroids and immunosuppressants may interfere T-cell responses towards MTB antigens [13, 32–34]. Therefore, immunosuppressive therapy may also influence the efficacy of the FluoroSpot assay. In our study, one patient with rheumatoid arthritis (RA) were taking prednisone (at 15 mg/d) when tested for both T-SPOT.TB and FluoroSpot. Both results of the RA patients were positive and similar for overall study population. Although it is highly possible that immunosuppressive therapy did not interfere the results of the study, further research on the potential influence of the immunosuppressive therapy is needed.

Both our study and previous research found that a number of iSFCs secreting TNF-α in the nil control of some samples were detected, but no iSFCs secreting IFN-γ were found [28]. A possible reason was that these spots formed without antigen stimulation may be caused by TNF-producing monocytes or dendritic cells instead of lymphocytes. TNF-α, a monokine involved in innate immune system, was mainly secreted by activated monocytes and macrophages, yet activated dendritic cells, γδT cells, CD4 + T cells and CD8 + T cells can also produce TNF-α [16]. In addition to lymphocytes, the PBMC for FluoroSpot testing also included monocytes and DC cells, and the innate immune response in which they participated also produced TNF-α, causing background spots in the nil control without MTB specific antigen stimulation.

However, this study has some limitations. First, we excluded patients with malignancy or undergoing TNF-α antagonist therapy, whose TNF-α secretion may be disturbed. Thus, whether IFN-γ/TNF-α FluoroSpot can be applied to these population required further research. Second, the use of T-SPOT.TB as a diagnostic criterion for LTBI may lead to selection bias [35]. However, IGRA is one of the recognized methods for detecting LTBI with satisfying specificity, it is reasonable to reduce the risk of misdiagnosis in the control group. Third, the overlapping in cytokines responses still exist, which may affect the correct distinction between groups. The high background in the nil control when detecting TNF-α should be concerned, which may affect the results. Forth, this was only a preliminary study of diagnostic test with case-control design, making overestimation of the diagnostic accuracy highly possible, and the results need to be further verified by cross-sectional or prospective cohort studies.

## Conclusion

IFN-γ/TNF-α FluoroSpot assay might be helpful for the differential diagnosis of ATB and LTBI. The frequencies of ESAT-6-specific total TNF-α-secreting T cells might be a better potential marker for IFN-γ/TNF-α FluoroSpot assay, compared with IFN-γ-secreting T cells. The results need to be further verified by cross-sectional or prospective cohort studies.

## Supplementary Information

**Additional file 1 Supplementary Table 1**. Frequencies of single TNF-α-, total TNF-α-, single IFN-γ-, total IFN-γ-, dual IFN-γ/TNF-α-secreting T cells following ESAT-6 or/and CFP-10 stimulation in ATB, LTBI and HC groups. **Supplementary Table 2**. Frequencies of spot-forming T cell in nil control in ATB, LTBI and HC groups. **Supplementary Table 3**. Diagnostic accuracy of single TNF-α-, total TNF-α-, single IFN-γ-, total IFN-γ-, dual IFN-γ/TNF-α-secreting T cells following ESAT-6 or/and CFP-10 stimulation in differentiating ATB and LTBI.

## Data Availability

The datasets used and analysed during the current study are available from the corresponding author on reasonable request.

## Abbreviations

ATB: active tuberculosis
LTBI: latent tuberculosis infection
TB: tuberculosis
MTB: *mycobacterium. Tuberculosis*
TST: tuberculin skin test
IGRA: interferon-gamma release assay
PPD: purified protein derivative
BCG: Bacille Calmette–Gue’rin
TNF: tumor necrosis factor
NAA: nucleic acid amplification
HC: healthy control
PBMCs: peripheral blood mononuclear cells
AIM-V: A serum free cell culture medium, Gibco™ AIM V Medium liquid, Invitrogen, USA
ESAT-6: 6-kDa early-secreted antigenic target
CFP-10: 10-kDa culture filtrate protein
iSFCs: immunofluorescence spot forming cells
SFCs: spot forming cells
SD: standard deviation
IQR: interquartile range
CIs: confidence intervals
ROC curves: receiver operating characteristic curves
AUROC: areas under the ROC curves
PV: predictive value
LR: likelihood ratio
AFB: acid-fast bacilli
TARA: TNF-α-release assay
